# Neonatal Injury at Cephalic Vaginal Delivery: A Retrospective Analysis of Extent of Association with Shoulder Dystocia

**DOI:** 10.1371/journal.pone.0104765

**Published:** 2014-08-21

**Authors:** Cantekin Iskender, Oktay Kaymak, Kudret Erkenekli, Emin Ustunyurt, Dilek Uygur, Halil Ibrahim Yakut, Nuri Danisman

**Affiliations:** 1 Department of Perinatology, Dr Zekai Tahir Burak Research and Training Hospital, Ankara, Turkey; 2 Department of Obstetrics and Gynecology, Bursa Şevket Yılmaz Research and Education Hospital, Bursa, Turkey; 3 Department of Neonatology, Dr Zekai Tahir Burak Research and Training Hospital, Ankara, Turkey; University of Tennessee Health Science Center, United States of America

## Abstract

**Purpose:**

To describe the risk factors and labor characteristics of Clavicular fracture (CF) and brachial plexus injury (BPI); and compare antenatal and labor characteristics and prognosis of obstetrical BPI associated with shoulder dystocia with obstetrical BPI not associated with shoulder dystocia.

**Methods:**

This retrospective study consisted of women who gave birth to an infant with a fractured clavicle or BPI between January 2009 and June 2013. Antenatal and neonatal data were compared between groups. The control group (1300) was composed of the four singleton vaginal deliveries that immediately followed each birth injury. A multivariable logistic regression model, with backward elimination, was constructed in order to find independent risk factors associated with BPI and CF. A subgroup analysis involved comparison of features of BPI cases with or without associated shoulder dystocia.

**Results:**

During the study period, the total number of vaginal deliveries was 44092. The rates of CF, BPI and shoulder dystocia during the study period were 0,6%, 0,16% and 0,29%, respectively. In the logistic regression model, shoulder dystocia, GDM, multiparity, gestational age >42 weeks, protracted labor, short second stage of labor and fetal birth weight greater than 4250 grams increased the risk of CF independently. Shoulder dystocia and protracted labor were independently associated with BPI when controlled for other factors. Among neonates with BPI whose injury was not associated with shoulder dystocia, five (12.2%) sustained permanent injury, whereas one neonate (4.5%) with BPI following shoulder dystocia sustained permanent injury (p = 0.34).

**Conclusion:**

BPI not associated with shoulder dystocia might have a higher rate of concomitant CF and permanent sequelae.

## Introduction

Clavicular fracture (CF) during delivery is a relatively common complication of labor and delivery. The prevalence of neonatal CF varies from 0.2 to 2.3% [1]–[6]. To a large extent, CF is unpredictable and unpreventable, as most cases occur following uncomplicated vaginal deliveries [1], [2], [5]. Several risk factors for CF have been identified in the literature, including fetal macrosomia [1]–[8], shoulder dystocia [3], [7], [9], [10], gestational diabetes mellitus (GDM)[1], oxytocin use to augment labor [2], operative delivery [9], prolonged second stage of labor [4] and meconium staining of amniotic fluid [1]. Among these factors, only increased fetal birth weight has been consistently associated with neonatal CF. The association between shoulder dystocia and CF is somewhat inconsistent. While documented shoulder dystocia has been reported to have a strong association with CF in some studies [4], [7], other studies have not reached the same conclusion [1], [6]. This disparity has been attributed to the inherent subjectivity of the clinician’s definition and differences in self- reporting of the shoulder dystocia [1], [8].

Most neonates with CF have an uncomplicated course, with no significant comorbidites [1], [6]. However, some neonates may sustain brachial plexus injury (BPI), particularly when there is concomitant shoulder dystocia. BPI is believed to share the same pathophysiologic mechanism with CF [4] and similar to CF, many cases of BPI occur in uncomplicated deliveries [9]. Therefore, it has been suggested that both CF and BPI can occur without apparent shoulder dystocia [11]. BPI in the absence of shoulder dystocia is believed to have distinctive features such as higher rate of concomitant CF, and more frequent involvement of the posterior shoulder at delivery [11], [12]. It has also been suggested that BPI not related to shoulder dystocia has a worse prognosis than BPI associated with shoulder dystocia in terms of permanent neurologic sequelae [11], [12].

The purpose of this retrospective study was (1) to describe the risk factors for CF; (2) to describe the risk factors for BPI; (3) and to compare antenatal and labor characteristics and prognosis of obstetrical BPI associated with shoulder dystocia with obstetrical BPI not associated with shoulder dystocia.

## Materials and Methods

This retrospective study consisted of women who gave birth vaginally to an infant with a fractured clavicle or BPI at Dr Zekai Tahir Burak Research and Training Hospital, Ankara, Turkey, between January 2009 and June 2013. The patients were allocated to one of three groups: Group 1 consisted of patients with CF, Group 2 consisted of patients with BPI, and Group 3 was the control group.

The diagnosis of a fractured clavicle at our institution was made by the pediatricians. Clavicle X-rays were performed to confirm the diagnosis when CF was suspected. BPI was diagnosed in infants with absent or unequal Moro reflex and inability to rotate the arm externally, supinate the forearm, and abduct the shoulder. Seven CF cases occurred during cesarean delivery were excluded from the analysis.

The following maternal and labor characteristics were obtained by reviewing the patients’ medical records: maternal age, parity, gestational age at delivery, labor duration, use of induction of labor or epidural analgesia, presence of GDM, meconium staining of amniotic fluid, dysfunctional labor, and shoulder dystocia. The following neonatal data were obtained: fetal height, fetal weight, infant sex, fetal anomalies, and presence of BPI. The next four women with singleton pregnancies to deliver vaginally after each patient in the study group were selected for the control group.

All identified cases of neonatal BPI were followed up on an outpatient basis by physiotherapists at our institution. The neonates received standard neonatal physiotherapy, which included passive stretching, limb positioning, and tactile stimulation. Those with persistent neurologic deficits after one year of follow up were considered to have permanent BPI.

The diagnosis of shoulder dystocia was made when there was a delay of 60 seconds or more between the delivery of the head and that of the body or when ancillary maneuvers other than gentle downward traction to effect delivery were used [13]. Deliveries occurring prior to 37 weeks of gestation were recorded as preterm deliveries. Slower than normal labor (protraction disorders) or complete cessation of progress (arrest disorders) in the active phase of labor were defined as active phase disorders [14].

The study was approved by ethics and educational issues coordinating committee of Dr Zekai Tahir Burak research and training hospital. All of the patients have signed a written consent that their data can be used with appropriate ethical committee approval prior to hospital admission. Patient records/information was anonymized and de-identified prior to analysis. Statistical analysis was performed using SPSS version 18 (Statistical Package for the Social Sciences, Chicago, IL). One-way analysis of variance was performed for parametric variables between groups that had a normal distribution. The Kruskal–Wallis test was used to compare continuous variables that did not distribute normally. The Bonferroni correction was used as a post hoc test in pairwise comparisons between groups. A Chi-square test was performed for nominal or ordinal variables between groups. A multivariable logistic regression model, with backward elimination, was constructed in order to find independent risk factors associated with BPI and CF. P values less than 0.05 were considered significant.

## Results

**Figure 1:**
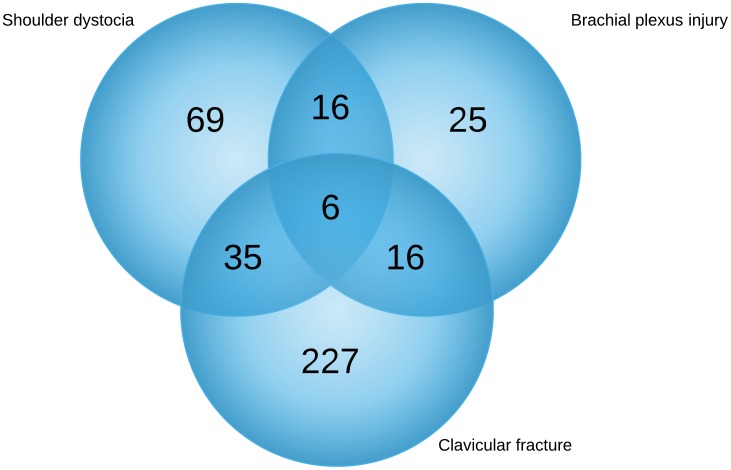
The incidence rates of Clavicular fracture, brachial plexus injury and shoulder dystocia.

**Figure 2:**
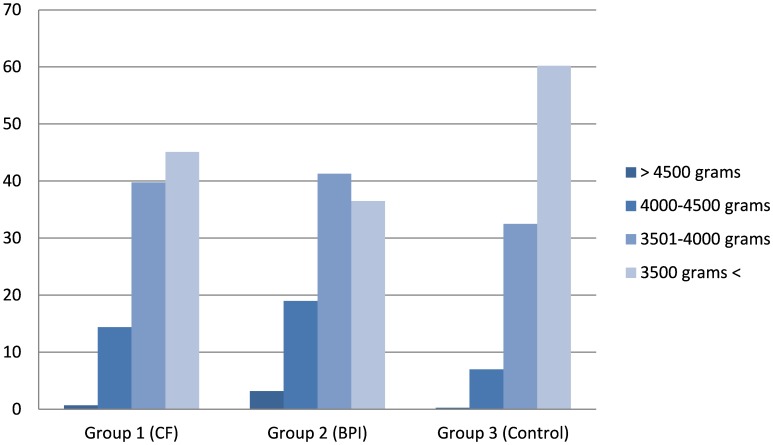
Neonatal birth weights of the groups with and without neonatal injury.

The total number of vaginal deliveries during the study period were 44092. During this period there were 71 instrumental vaginal deliveries (0.16% of all vaginal deliveries) and the cesarean delivery rate was 45.9%. The incidence rates of BPI, CF, and shoulder dystocia are shown in Figure 1; these include isolated cases as well as concurrent cases. During the study period, there were 227 isolated CF (0,5%),25 isolated BPI (0,06%) and 69 isolated shoulder dystocia (0,16%) cases occured. CF and concurrent shoulder dystocia occurred in 35 patients, while CF and concurrent BPI occurred in 16 patients. Both shoulder dystocia and BPI occurred together in six patients with CF. The rates of CF, BPI and shoulder dystocia during the study period were 0,6%, 0,16% and 0,29%, respectively. The neonatal birth weights of the groups with and without neonatal injury are shown in Figure 2. The mean birth weights of the two neonatal injury groups were higher than that of the control group (p<0.05; Table 1). The recorded maternal and antenatal characteristics are shown in Table 1. Maternal age at delivery, pre-pregnancy BMI, and rate of multiparity were similar among the groups. The rate of gestational diabetes was higher in the study groups than in the control group; maternal weight gain was also higher in the study groups than in the control group. Patients in the study groups delivered at a more advanced gestational age than those in the control group, and the rate of preterm deliveries was lower in the study groups than in the control group.

**Table 1:** Comparison of maternal, perinatal and neonatal characteristics.

	Group 1 (CF) (n = 284)	Group 2 (BPI) (n = 63)	Group 3 (Control group)(n = 1300)	P Groups		
				1vs3	2vs3	1vs2
Maternal age (years)	27,3±5,69	26,6±6,23	27,8±5,61	NS		
Parity				NS		
0	112 (39,4%)	29 (46,0%)	562 (43,2%)			
1–3	170 (59,9%)	33 (52,4%)	719 (55,3%)			
≥4	2 (0,7%)	1 (1,6%)	19 (1,5%)			
Weight gain (kilograms)	13,4±4,32	13,9±4,57	12,6±4,21	0,02	0,02	NS
Pre-pregnancy BMI	24,5±3,96	24,6±3,71	24,0±3,94	NS		
Gestational age (weeks)	39,1±1,8	39,1±1,7	38,3±2,2	<0,01	<0,01	NS
Gestational age>42 weeks	12 (4,2%)	3 (4,8%)	25 (1,9%)	0,02	NS	NS
Preterm delivery	14 (4,9%)	3 (4,8%)	160 (12,3%)	<0,01	NS	NS
1st stage duration (min)	265±126	259±146	236±104	<0,01	0,01	NS
2nd stage duration (min)	21,3±15,9	27,3±19,3	24,7±12,3	<0,01	<0,01	NS
Fetal weight(grams)	3536±435	3658±458	3309±588	<0,01	<0,01	<0,01
Fetal height (centimeters)	50,8±1,74	50,8±1,61	50,1±2,33	<0,01	<0,01	NS

Data expressed as number (%), mean ± SD, CF: Clavicular fracture, BPI: Brachial plexus injury, NS: non significant, min: minutes.

The risk factors for CF are shown in Table 2. The unadjusted odds ratios for CF were significant for shoulder dystocia, fetal birth weight greater than 4000 grams, GDM, multiparity and post-term pregnancy, protracted labor, and a short second stage of labor. In the logistic regression model, all of these variables, except fetal birth weight greater than 4000 grams, were also independently associated with CF. Fetal birth weight greater than 4250 grams also increased the risk of CF independently. The risk factors for BPI are shown in Table 2. Shoulder dystocia, and protracted labor were independently associated with BPI when controlled for other factors. Table 3 provides a comparison of neonatal BPI with and without concomitant shoulder dystocia. Five neonates (12.2%) with BPI not associated with shoulder dystocia sustained permanent injury, whereas one neonate (4.5%) with BPI following shoulder dystocia sustained permanent injury (p = 0.34). Other antepartum and intrapartum characteristics, including concomitant CF, were similar.

**Table 2:** Risk factors for CF and BPI.

Characteristics	Adjusted Odds ratio (95% CI)	
	BPI	CF
Shoulder dystocia	59,8 (22,9–156,6)*	35,3 (14,4–86,6)*
Birth weight>4250 grams	3,5 (0,7–17,8)	4,1 (1,4–11,8)*
Birth weight>4000 grams	1,2 (0,4–3,8)	1,3 (0,8–2,2)
GDM	1,2 (0,4–3,9)	2,8 (1,7–4,5)*
Multiparity	1,0 (0,5–1,7)	1,3 (1,0–1,8)*
Male fetus	1,5 (0,8–2,7)	0,9 (0,7–1,2)
Gestational age>42 weeks	3,1 (0,8–12,5)	2,3 (1,1–5,0)*
Meconium staining	0,5 (0,2–1,3)	0,7 (0,5–1,1)
Oxytocin use during labor	0,7 (0,4–1,4)	0,8 (0,6–1,0)
Protracted labor	2,5 (1,1–5,9)*	1,6 (1,0–2,5)*

CF: Clavicular fracture, BPI: Brachial plexus injury, CI: confidence interval, GDM: Gestational diabetes mellitus, * p<0.05.

**Table 3:** Comparison of features of BPI cases with or without associated shoulder dystocia.

	BPI without Shoulder dystocia (n = 41) (%)	BPI with Shoulder dystocia (n = 22) (%)	p
Clavicular fracture	16 (39%)	6 (27,3%)	0,35
Permanent BPI	5 (12,2%)	1 (4,5%)	0,34
Gestational diabetes	7 (17,1%)	4 (18,2%)	0,72
Fetal weight>4250 grams	4 (9,8%)	3 (13,6%)	0,64
Fetal weight>4000 grams	8 (14,5%)	6 (27,3%)	0,48
Male Fetus	18 (43,0%)	8 (36,4%)	0,56

Data expressed as number (%), BPI: Brachial plexus injury.

## Discussion

Certain labor characteristics and intrapartum complications differed between the patients with neonatal injury and the control group. These factors include a greater weight gain in pregnancy, longer gestational age and a higher incidence of post-term pregnancy in the groups with CF or BPI (Groups 1 and 2). The neonates in groups 1 and 2 were longer and heavier than the neonates in the control group. These findings are to be expected, as all of the mentioned factors are associated with shoulder dystocia and consequent neonatal trauma. As shown in Table 2, when controlled for confounding factors, independent predictors of CF were shoulder dystocia, fetal birth weight greater than 4250 grams, GDM, multiparity, post-term pregnancy, protracted labor and short second stage of labor. Independent predictors of BPI were shoulder dystocia and protracted labor.

Previous data clearly indicates that a certain percentage of neonatal injury cases are associated with shoulder dystocia [1], [4], [7], [9], [15]–[17]. Data regarding the association between BPI and shoulder dystocia is consistent [4], [9], [15]–[17], while the association between shoulder dystocia and CF is more controversial. Some studies [3], [7], [9], [10] have found a clear association between CF and shoulder dystocia, while others [1], [6], [18] have failed to detect such an association. However, the sample sizes of the studies that failed to detect an association between CF and shoulder dystocia were small. In addition, the overall rate of shoulder dystocia was either too high (around 18% in one study [1]) or too low (0% in two studies [6], [18]). Our results are clearly in agreement with those of studies that detected an association between shoulder dystocia and CF, as shoulder dystocia occurred in 14% of our patients with CF.

As stated above, one of the main results of this study is that shoulder dystocia is the major factor that causes both CF and BPI. However, the interrelation among BPI, CF, and shoulder dystocia is difficult to interpret from our data. Some patients with shoulder dystocia had BPI but not CF, while others had the opposite. In a few cases, both conditions occurred together. There have been numerous previous attempts to explain this relationship. It was initially suggested that deliveries associated with neonatal injury (particularly BPI) were actually shoulder dystocia cases that were not recognized [19]. However, this idea has been proven wrong by solid evidence, as several studies, including ours, have reported cases of neonatal injury not associated with shoulder dystocia [9], [11], [12], [15]–[17].

Another theory aimed to differentiate BPI cases not associated with shoulder dystocia [12]. This was initially suggested by Gherman and colleagues, who stated that factors other than shoulder dystocia might play a role in BPI [11]. In their study, the authors stressed distinct features of BPI not related to shoulder dystocia, such as a higher rate of concomitant CF. Referring to earlier experimental models [19], [20], the authors have postulated that use of the McRoberts maneuver in cases of shoulder dystocia increased the anteroposterior diameter of the pelvic outlet and offered protection against CF. However, in other studies, including the authors’ later work [7], , it has not been confirmed that the McRoberts maneuver prevents neonatal BPI and CF in cases of shoulder dystocia.

In more recent studies, the authors suggested that BPI not related to shoulder dystocia had a higher rate of concomitant CF, permanent neurologic sequelae, and more frequent involvement of the posterior shoulder at delivery [12]. These suggestions were strongly criticized by others, who contended that they were based on indirect evidence provided by retrospective data collected from small samples [22]. In addition, some of the projections of this theory, such as more frequent involvement of the posterior shoulder or higher rate of concomitant CF, could not be reproduced in other studies [16]. Our data showed that BPI cases that occurred in the absence of shoulder dystocia had a higher rate of concomitant CF. Moreover, among six neonates with permanent BPI, five were not related to shoulder dystocia. While these observations support the theory, none reached statistical significance. We were unable to provide data regarding the involvement of the posterior shoulder.

When our results are collectively considered from a pathophysiologic perspective, it can be stated that shoulder dystocia is the major determinant of BPI, whereas many other antepartum and intrapartum factors can independently lead to CF. Interestingly, many of these factors are also risk factors for shoulder dystocia. A possible explanation, as discussed previously, is underreporting of shoulder dystocia. There have been active efforts to properly recognize and manage shoulder dystocia at our institution since 2009. These efforts include a review of shoulder dystocia cases with a consultant perinatologist on a daily basis and archiving them separately. However, in a study with a retrospective design, we can never be certain that all cases were objectively reported.

Our study has several limitations. First, the retrospective design of the study might limit the validity of the findings, for reasons that are inherent in all retrospective studies. The high rate of cesarean sections and avoidance of operative delivery at our institution might have an impact on risk factors for CF and BPI. In addition, reliable data on anterior or posterior shoulder involvement were not available. To the best of our knowledge, however, our study is among the few that have aimed to analyze the relationship among three significant labor complications.

In conclusion, the findings of the present study suggest that not all CF or BPI cases are caused by shoulder dystocia. In addition, BPI not associated with shoulder dystocia had a higher rate of concomitant CF and long-term sequelae, but these results did not reach statistical significance. These findings led us to believe that intrapartum factors other than shoulder dystocia might play a role in neonatal birth injury at cephalic presentation. This issue certainly merits investigation using prospective studies with careful designs, as the evidence for neonatal injury not related to shoulder dystocia is scarce and subject to contradiction.

## References

[1] Beall MH, Ross MG (2001) Clavicle fracture in labor: risk factors and associated morbidities. J Perinatol 21: 513–515. 11774010 10.1038/sj.jp.7210594

[2] Chez RA, Carlan S, Greenberg SL, Spellacy WN (1994) Fracture clavicle is an unavoidable event. Am J Obstet Gynecol 171: 797–798. 8092231 10.1016/0002-9378(94)90100-7

[3] Kaplan B, Rabinerson D, Avrech OM, Carmi N, Steinberg DM, et al (1998) Fracture of the clavicle in the newborn following normal labor and delivery. Int J Gynaecol Obstet 63: 15–20. 9849706 10.1016/s0020-7292(98)00127-1

[4] Peleg D, Hasnin J, Shalev E (1997) Fractured clavicle and Erb’s palsy unrelated to birth trauma. Am J Obstet Gynecol 177: 1038–1040. 9396889 10.1016/s0002-9378(97)70010-3

[5] Roberts SW, Hernandez C, Maberry MC, Adams MD, Leveno KJ, et al (1995) Obstetric clavicular fracture: the enigma of normal birth. Obstet Gynecol 86: 978–981. 7501352 10.1016/0029-7844(95)00277-X

[6] Lurie S, Wand S, Golan A, Sadan O (2011) Risk factors for fractured clavicle in the newborn. J Obstet Gynaecol Res 37: 1572–1574. 21790882 10.1111/j.1447-0756.2011.01576.x

[7] Gherman RB, Ouzounian JG, Goodwin TM (1998) Obstetric maneuvers for shoulder dystocia and associated neonatal morbidity. Am J Obstet Gynecol 178: 1126–1130. 9662290 10.1016/s0002-9378(98)70312-6

[8] Gherman RB, Chauhan S, Ouzounian JG, Lerner H, Gonik B, et al (2006) Shoulder dystocia: the unpreventable obstetric emergency with empiric management guidelines. Am J Obstet Gynecol 195: 657–672. 16949396 10.1016/j.ajog.2005.09.007

[9] Walsh JM, Kandamany N, Ni Shuibhne N, Power H, Murphy JF, et al (2011) Neonatal brachial plexus injury: comparison of incidence and antecedents between 2 decades. Am J Obstet Gynecol 204: 324.e1–6. 21345417 10.1016/j.ajog.2011.01.020

[10] Lam MH, Wong GY, Lao TT (2002) Reappraisal of neonatal clavicular fracture: relationship between infant size and neonatal morbidity. Obstet Gynecol 100: 115–119. 12100812 10.1016/s0029-7844(02)02055-0

[11] Gherman RB, Ouzounian JG, Miller DA, Kwok L, Goodwin TM (1998) Spontaneous vaginal delivery: a risk factor for Erb’s palsy? Am J Obstet Gynecol 178: 423–427. 9539501 10.1016/s0002-9378(98)70413-2

[12] Sandmire HF, DeMott RK (2002) Erb’s palsy without shoulder dystocia. Int J Gynaecol Obstet 78: 253–256. 12384274 10.1016/s0020-7292(02)00131-5

[13] Beall MH, Spong C, McKay J, Ross MG (1998) Objective definition of shoulder dystocia: a prospective evaluation. Am J Obstet Gynecol 179: 934–937. 9790373 10.1016/s0002-9378(98)70191-7

[14] American College of Obstetricians and Gynecologists: Dystocia and augmentation of labor. Practice Bulletin No. 49, December 2003.

[15] Perlow JH, Wigton T, Hart J, Strassner HT, Nageotte MP, et al (1996) Birth trauma: a five-year review of incidence and associated perinatal factors. J Reprod Med 41: 754–760. 8913978

[16] Chauhan SP, Rose CH, Gherman RB, Magann EF, Holland MW, et al (2005) Brachial plexus injury: a 23-year experience from a tertiary center. Am J Obstet Gynecol 192: 1795–1800. 15970811 10.1016/j.ajog.2004.12.060

[17] Gilbert WM, Nesbitt TS, Danielsen B (1999) Associated factors in 1611 cases of brachial plexus injury. Obstet Gynecol 93: 536–540. 10214829 10.1016/s0029-7844(98)00484-0

[18] Hsu TY, Hung FC, Lu YJ, Ou CY, Roan CJ, et al (2002) Neonatal clavicular fracture: clinical analysis of incidence, predisposing factors, diagnosis, and outcome. Am J Perinatol 19: 17–21. 11857092 10.1055/s-2002-20169

[19] Gonik B, Allen R, Sorab J (1989) Objective evaluation of the shoulder dystocia phenomenon: effect of maternal pelvic orientation on force reduction. Obstet Gynecol 74: 44–48. 2733940

[20] Allen R, Sorab J, Gonik B (1991) Risk factors for shoulder dystocia: an engineering study of clinician-applied forces. Obstet Gynecol 77: 352–355. 1992397

[21] Leung TY, Stuart O, Suen SS, Sahota DS, Lau TK, et al (2011) Comparison of perinatal outcomes of shoulder dystocia alleviated by different type and sequence of manoeuvres: a retrospective review. BJOG 118: 985–990. 21481159 10.1111/j.1471-0528.2011.02968.x

[22] Allen RH, Edelberg SC (2003) Brachial plexus palsy causation. Birth 30: 141–143; author reply 143–145. 10.1046/j.1523-536x.2003.00236_2.x 12752176

